# Supplementary material for: An extension of the Walsh-Hadamard transform to calculate and model epistasis in genetic landscapes of arbitrary shape and complexity
Source: PLoS Comput Biol. 2024 May 28;20(5):e1012132. doi: 10.1371/journal.pcbi.1012132 (PMC11161127; doi:10.1371/journal.pcbi.1012132)
Supplement: S1 Text — (PDF) [file pcbi.1012132.s003.pdf]

S1 Text. Supplementary Methods

Here we provide the proofs of the mathematical results shown in the main text.

Proposition 1. *Let us define the matrices A_n recursively as*

$$A_{n+1} = \frac{1}{s}\begin{pmatrix} A_n & A_n & A_n & \dots & A_n \\ A_n & (1-s)A_n & A_n & \dots & A_n \\ A_n & A_n & (1-s)A_n & \ddots & \vdots \\ \vdots & \vdots & \ddots & \ddots & A_n \\ A_n & A_n & \dots & A_n & (1-s)A_n \end{pmatrix} \qquad A_0 = 1 \qquad \textit{and} \qquad A_n \ \textit{is} \ s^n \times s^n. \tag{13}$$

For $n \in \mathbb{N}$, A_n is the inverse of the matrix H_n defined in equation (10).

Proof. Let us prove this by induction. For $n = 1$ we have

$$H_1 = \begin{pmatrix} 1 & 1 & 1 & \dots & 1 \\ 1 & -1 & 0 & \dots & 0 \\ 1 & 0 & -1 & \ddots & \vdots \\ \vdots & \vdots & \ddots & \ddots & 0 \\ 1 & 0 & \dots & 0 & -1 \end{pmatrix} \tag{26}$$

$$A_1 = \frac{1}{s}\begin{pmatrix} 1 & 1 & 1 & \dots & 1 \\ 1 & 1-s & 1 & \dots & 1 \\ 1 & 1 & 1-s & \ddots & \vdots \\ \vdots & \vdots & \ddots & \ddots & 1 \\ 1 & 1 & \dots & 1 & 1-s \end{pmatrix}. \tag{27}$$

The rows and columns of these two matrices can be described as follows:

$$(H_1)_{i\cdot} = \begin{cases} (1, 1, \dots, 1) & \text{if } i = 1 \\ (1, 0, \dots, 0, h_i, 0, \dots, 0) & \text{if } i \neq 1, \end{cases}$$

$$(A_1)_{\cdot j} = \frac{1}{s}\begin{cases} (1, 1, \dots, 1)^T & \text{if } j = 1 \\ (1, 1, \dots, 1, a_j, 1, \dots, 1)^T & \text{if } j \neq 1, \end{cases}$$

where $h_i := (H_1)_{ii} = -1 \ \forall i > 1$ and $a_j := (A_1)_{jj} = 1 - s \ \forall j > 1$.

Therefore,

$$(H_1 \cdot A_1)_{ij} = (H_1)_{i\cdot} \cdot (A_1)_{\cdot j} = \frac{1}{s} \begin{cases} s & \text{if } i = j \\ 0 & \text{if } i \neq j \end{cases}$$

$$H_1 \cdot A_1 = \frac{1}{s} \begin{pmatrix} s & 0 & \dots & 0 \\ 0 & s & \ddots & \vdots \\ \vdots & \ddots & \ddots & 0 \\ 0 & \dots & 0 & s \end{pmatrix} = I_{s \times s},$$

where $I_{s\times s}$ is the identity matrix of size $s \times s$. Since both H_1 and A_1 are symmetric, it is also true that $A_1 \cdot H_1 = I_{s\times s}$. Therefore, A_1 is the inverse of H_1.

Assume that the hypothesis is true for a fixed $n \in \mathbb{N}$. Let us now prove that it is also true for $n+1$. Following the recursive definitions of H_{n+1} and A_{n+1} in equations (10) and (13), we can write the blocks of these matrices as follows:

$$(H_{n+1})_{[i][\cdot]} = \begin{cases} (H_n, H_n, \dots, H_n) & \text{if } i = 1 \\ (H_n, 0, \dots, 0, \tilde{h}_i, 0, \dots, 0) & \text{if } i \neq 1, \end{cases} \tag{28}$$

where $(H_{n+1})_{[i][j]}$ denotes the block at position i, j in H_{n+1} and $\tilde{h}_i := (H_{n+1})_{[i][i]} = -H_n \ \forall i > 1$;

$$(A_{n+1})_{[\cdot][j]} = \frac{1}{s} \begin{cases} (A_n, A_n, \dots, A_n)^T & \text{if } j = 1 \\ (A_n, A_n, \dots, A_n, \tilde{a}_j, A_n, \dots, A_n)^T & \text{if } j \neq 1, \end{cases} \tag{29}$$

where $(A_{n+1})_{[i][j]}$ denotes the block at position i, j in A_{n+1} and $\tilde{a}_j := s(A_{n+1})_{[j][j]} = (1-s)A_n \ \forall j > 1$. We can therefore write the block at position i, j of the product of these matrices as follows:

$$(H_{n+1} \cdot A_{n+1})_{[i][j]} = (H_{n+1})_{[i][\cdot]} \cdot (A_{n+1})_{[\cdot][j]} = \frac{1}{s} \begin{cases} sH_n \cdot A_n & \text{if } i = j \\ 0 & \text{if } i \neq j. \end{cases}$$

According to the induction hypothesis we know that H_n and A_n are inverse matrices i.e. $H_n \cdot A_n = I_{s^n \times s^n}$. Therefore, the blocks on the diagonal are identity matrices and the blocks outside the diagonal are zeros. This means that $H_{n+1} \cdot A_{n+1} = I_{s^{n+1} \times s^{n+1}}$. Similarly, due to the symmetry of the matrices we can also prove that $A_{n+1} \cdot H_{n+1} = I_{s^{n+1} \times s^{n+1}}$.

We can then conclude that $A_n = H_n^{-1}$ for every $n \in \mathbb{N}$. □

The proof above can be greatly simplified with the use of the Kronecker product and its properties as follows:

Proof. It is straightforward to observe that the recursive notation of H_{n+1} can be expressed as a Kronecker

product between H_1 and H_n. With this, we can use that the inverse of the Kronecker product is the product of the inverses of the individual matrices, if and only if, the individual matrices are invertible [1]. In our case, it is easy to see that the latter is true because we have shown that H_1 is invertible (we computed its inverse in the previous proof), and all the other matrices are compute as a Kronecker product that derive uniquely from H_1 (H_2 is invertible because $H_2 = H_1 \otimes H_1$, H_3 is invertible because $H_3 = H_1 \otimes H_2$, and so forth. Therefore,

$$A_{n+1} = (H_{n+1})^{-1} = (H_1 \otimes H_n)^{-1} = H_1^{-1} \otimes H_n^{-1} = A_1 \otimes A_n$$

Now, we only need to compute the inverse of H_1 as we did at the beginning of the original proof, and we obtain the recurrent definition of A_{n+1} (13) by applying the Kronecker product between A_1 as in (27) and A_n. □

Proposition 2. *The elements of H_n can be written as*

$$(H_n)_{ij} = \begin{cases} (-1)^{(E_n)_{ij}} & \textit{if } (M_n)_{ij} = n \\ 0 & \textit{otherwise,} \end{cases}$$

where M and E are $s^n \times s^n$ matrices whose elements are

$$(E_n)_{ij} = \sum_{\substack{k=1 \\ i_k \cdot j_k > 0}}^{n} \delta_{i_k j_k} \tag{15}$$

$$(M_n)_{ij} = \sum_{\substack{k=1 \\ i_k \cdot j_k > 0}}^{n} \delta_{i_k j_k} + \sum_{\substack{k=1 \\ i_k \cdot j_k = 0}}^{n} 1 = (E_n)_{ij} + \sum_{\substack{k=1 \\ i_k \cdot j_k = 0}}^{n} 1,$$

where δ_{ij} denotes the Kronecker delta of i, j.

Proof. Let us prove the formula by induction. For $n = 1$ and any given s, H_n is given by equation (26). Therefore, we can write $(E_1)_{ij}$ and $(M_1)_{ij}$ as follows:

$$(M_1)_{ij} = \begin{cases} 1 & \text{if } i = 1 \text{ or } j = 1 \\ 1 & \text{if } i = j \\ 0 & \text{if } i \neq j \neq 1 \end{cases}$$

$$(E_1)_{ij} = \begin{cases} 1 & \text{if } i = j \neq 1 \\ 0 & \text{otherwise.} \end{cases} \tag{30}$$

Therefore, since $n = 1$, $(M_1)_{ij} = n = 1$ only when either $i = 1$, $j = 1$ or $i = j$. In the rest of the cases $(M_1)_{ij} \neq n = 1$ and, according to the formula, the elements of the matrix will be 0. Now, for the cases where $(M_1)_{ij} = n = 1$, we need to check the value of $(E_1)_{ij}$. We can see how $(E_1)_{ij} = 1$ only when $i = j$ and they are different from 1. This means that all the elements of the diagonal of H_1, except the first one, will be $(-1)^1 = -1$ and the first row and first columns will have $(-1)^0 = 1$. The rest of the elements correspond to $(M_1)_{ij} \neq n = 1$ so they will be filled with zeros. Putting all this together, we find the expression as H_1 from equation (26).

Assume now that the expression is true for a fixed $n \in \mathbb{N}$ and let us prove it for $n+1$. In this case, the matrix of H_{n+1} is defined by blocks (see equation (10)). We first define the indices $P \in \{1, \dots, s\}$ and $Q \in \{1, \dots, s\}$ for row and column blocks, respectively. The first matrix block of H_{n+1} corresponds to $P = 1$ and $Q = 1$. The corresponding blocks of the matrices M_{n+1} and E_{n+1}, which are necessary for the derivation of the block in H_{n+1}, are computed by comparing the genotype sequences $\vec{p}, \vec{q} \in \mathcal{S}^{n+1}$ for which $\vec{p} = (0, i_1, \dots, i_n) := 0 \frown \vec{i}$ for $\vec{i} \in \mathcal{S}^n$ and $\vec{q} = 0 \frown \vec{j}, \vec{j} \in \mathcal{S}^n$. More generally, the block in the Pth position with respect to the rows and Q^{th} position with respect to the columns can be obtained by comparing the genotype sequences $\vec{p}, \vec{q} \in \mathcal{S}^{n+1}$ for which $\vec{p} = (P-1) \frown \vec{i}, \vec{i} \in \mathcal{S}^n$ and $\vec{q} = (Q-1) \frown \vec{j}, \vec{j} \in \mathcal{S}^n$. See below for a visual description of the notation.

From these observations, it can easily be deduced that for any $\vec{p} = (P-1) \frown \vec{i}, \vec{q} = (Q-1) \frown \vec{j}$ with $\vec{i}, \vec{j} \in \mathcal{S}^n$, $P-1, Q-1 \in \mathcal{S}$,

$$(M_{n+1})_{pq} = \begin{cases} (M_n)_{ij} + 1 & \text{if } P = 1 \text{ or } Q = 1 \\ (M_n)_{ij} + 1 & \text{if } P = Q \\ (M_n)_{ij} & \text{if } P \neq Q \neq 1 \end{cases} \tag{31}$$

$$(E_{n+1})_{pq} = \begin{cases} (E_n)_{ij} + 1 & \text{if } P = Q \neq 1 \\ (E_n)_{ij} & \text{otherwise,} \end{cases} \tag{32}$$

where $i = p - s^n(P-1)$, $j = q - s^n(Q-1)$, $P = \lceil p/s^n \rceil$ and $Q = \lceil q/s^n \rceil$, with $\lceil . \rceil$ denoting the ceiling function. A visual description of the notation of the block structure of the matrices is given by

$$X_{n+1} = \text{row block } P\left\{\begin{pmatrix} X_n^{11} & \cdots & \overbrace{X_n^{1Q}}^{\text{column block } Q} & \cdots & X_n^{1s} \\ \vdots & \ddots & \vdots & & \vdots \\ X_n^{P1} & \cdots & X_n^{PQ} & \cdots & X_n^{Ps} \\ \vdots & & \vdots & \ddots & \vdots \\ X_n^{s1} & \cdots & \underset{\substack{\uparrow \\ \text{column } q}}{X_n^{sQ}} & \cdots & X_n^{ss} \end{pmatrix}\right. \leftarrow \text{row } p$$

Here X_{n+1} denotes any generic matrix following the structure of the matrices in equations (10), (11), (13), (31) and (32).

Now, similar to the case $n = 1$, we have that $(M_{n+1})_{pq} = n + 1$ only when $(M_n)_{ij} = n$ and either $P = 1$, $Q = 1$ or $P = Q$, which corresponds to the newly added state being $p_1 = P - 1 = 0$, $q_1 = Q - 1 = 0$ or $p_1 = q_1$. In the rest of the cases $(M_{n+1})_{pq} \neq n + 1$ and the elements of the matrix H_{n+1} will be 0. Now, for the cases where $(M_{n+1})_{pq} = n+1$, we will have that $(E_{n+1})_{pq}$ has either the same value of the corresponding entry in E_n or it will be increased by 1. This means, that when $P = Q \neq 1$, the sign of the entry in H_{n+1} will be inverted. Otherwise, the sign of the element of the matrix stays the same. With this we prove the formula for H_{n+1}, since we have shown how we can find the same block structure as in equation (10). By induction, we can conclude that the formula holds for every $n \in \mathbb{N}$. □

Proposition 3. *The elements of A_n can be written as*

$$(A_n)_{ij} = \frac{1}{s^n}(1 - s)^{(E_n)_{ij}}, \tag{17}$$

where E_n is defined as in equation (15).

Proof. Let us prove the formula by induction.

For any given s, A_1 is defined in equation 27. Its diagonal elements are equal to $(1 - s)/s$ for $i > 1$, $1/s$ for $i = 1$ and its off-diagonal elements are equal to $1/s$. It can easily be observed from equation (30) that the RHS of equation (17) is equal to $(1 - s)/s$ for $i = j \neq 1$ and to $1/s$ otherwise, so equation (17) is true for $n = 1$.

Assume now that equation (17) is true for a fixed $n \in \mathbb{N}$ and let us prove it for $n + 1$. We use the recursive definition of A_{n+1} in equation (13) and the block representation of the genotype sequences as in the proof of (2).

Let us start with the first block in the diagonal of A_{n+1}, i.e. $P = 1$ and $Q = 1$, where the entries of the corresponding block in E_{n+1} are derived from comparisons of pairs of genotype sequences of the form $\vec{p} = 0 \frown \vec{i}, \vec{q} = 0 \frown \vec{j}$ for $\vec{i}, \vec{j} \in \mathcal{S}^n$. From equation (13), this block is equal to $\frac{1}{s}A_n$, so writing $i = p \bmod s^n$ and $j = q \bmod s^n$, we have

$$(A_{n+1})_{pq} = \frac{1}{s}(A_n)_{ij} = \frac{1}{s^{n+1}}(1-s)^{(E_n)_{ij}}.$$

From equation (32), $(E_{n+1})_{pq} = (E_n)_{ij}$, which yields the desired result.

Now let us consider the elements in the other diagonal blocks of A_{n+1}, where the entries correspond to pairs of genotype sequences of the form $\vec{p} = (P-1) \frown \vec{i}, \vec{q} = (Q-1) \frown \vec{j}$ with $P = Q \neq 1$ and $\vec{i}, \vec{j} \in \mathcal{S}^n$. From equation (13), this block is equal to $\frac{1}{s}(1-s)A_n$, i.e.

$$(A_{n+1})_{pq} = \frac{1}{s}(1-s)(A_n)_{ij} = \frac{1}{s^{n+1}}(1-s)^{(E_n)_{ij}+1}.$$

From equation (32), $(E_{n+1})_{pq} = (E_n)_{ij} + 1$, which yields the desired result.

Finally, let us consider the elements in the off-diagonal blocks of A_{n+1}, where the entries correspond to pairs of genotype sequences of the form $\vec{p} = (P-1) \frown \vec{i}, \vec{q} = (Q-1) \frown \vec{j}$ with $P \neq Q$ and $\vec{i}, \vec{j} \in \mathcal{S}^n$. From equation (13), this block is equal to $\frac{1}{s}A_n$, so we have

$$(A_{n+1})_{pq} = \frac{1}{s}(A_n)_{ij} = \frac{1}{s^{n+1}}(1-s)^{(E_n)_{ij}}.$$

From equation (32), $(E_{n+1})_{pq} = (E_n)_{ij}$, which completes the proof. □

Proposition 4. *The matrices V_n and V_n^{-1} are diagonal matrices whose diagonal elements can be written as*

$$(V_n)_{ii} = (-1)^{n-W_n(\vec{i})}\frac{1}{s^{W_n(\vec{i})}} \tag{18}$$

and

$$(V_n^{-1})_{ii} = (-1)^{n-W_n(\vec{i})}s^{W_n(\vec{i})}, \tag{19}$$

where

$$W_n(\vec{i}) := \sum_{k=1}^{n} w_k, \textit{ with } w_k := \delta_{i_k 0}$$

and $\vec{i}$ again denotes the ith element in $\mathcal{S}^n$ when ordered by the base s representation of integers.

Proof. Let us prove equation (18) by induction, equation (19) follows directly.

One can easily check from equation (11) that the formula holds for $n = 1$. Let us now assume that equation (18) is true for a fixed $n \in \mathbb{N}$ and let us prove it for $n+1$. We use the recursive definition of V_{n+1} in equation (11). Let us consider the element $(V_{n+1})_{pp}$. If $\vec{p} = 0 \frown \vec{i}$, this corresponds to the first block of V_{n+1}, i.e. $P = 1$, where the elements are multiplied by $1/s$ and $W_{n+1}(\vec{p}) = W_n(\vec{i}) + 1$, so

$$(V_{n+1})_{pp} = \frac{1}{s}(V_n)_{ii} = (-1)^{n-W_n(\vec{i})}\frac{1}{s^{W_n(\vec{i})+1}} = (-1)^{n+1-W_{n+1}(\vec{p})}\frac{1}{s^{W_{n+1}(\vec{p})}}.$$

Similarly, if $\vec{p} = (P-1) \frown \vec{i}$ and $P > 1$, i.e. for the other diagonal blocks, from the recursive formula the elements are multiplied by -1 and $W_{n+1}(\vec{p}) = W_n(\vec{i})$, so by writing again $i = p - s^n(P-1)$, where $P = \lceil p/s^n \rceil$, we have

$$(V_{n+1})_{pp} = -(V_n)_{ii} = (-1)^{1+n-W_n(\vec{i})} \frac{1}{s^{W_n(\vec{i})}} = (-1)^{n+1-W_{n+1}(\vec{p})} \frac{1}{s^{W_{n+1}(\vec{p})}},$$

which completes the proof. □

Proposition 5. *The matrix A_n defined in equation (21) is the inverse of the matrix H_n in the general case where each position can have a different number of states.*

Proof. Let us prove by induction that $H_n \cdot A_n = I$ where I is the identity matrix of the corresponding size. Since H_n and A_n are symmetric, this would imply that $A_n \cdot H_n = I$ as well, and therefore, $A_n = H_n^{-1}$.

The case $n = 1$ corresponds exactly to the case $n = 1$ of the proof of Proposition 1 by setting $s = s_1$. Therefore, $H_1 A_1 = I_{s_1 \times s_1}$, and A_1 is the inverse of H_1.

Now, assume the hypothesis is true for a fixed $n \in \mathbb{N}$ and let us prove that this is also true for $n+1$. We can write the rows and columns of the matrices H_{n+1} and A_{n+1} as equation (28) and equation (29), respectively. The only difference is that we need to replace s by s_{n+1} and the size of the matrices is different. Following exactly the same derivation as in Proposition 1 we can conclude that $H_{n+1} A_{n+1} = I$ and this proves by induction that $A_n = H_n^{-1}$. □

Proposition 6. *In this general case, the elements of H_n and A_n can be written as*

$$(H_n)_{ij} = \begin{cases} (-1)^{(E_n)_{ij}} & \textit{if } (M_n)_{ij} = n \\ 0 & \textit{otherwise} \end{cases}$$

$$(A_n)_{ij} = \frac{\prod_{k=1}^{n} \left(1 - s_k\right)^{e_k}}{\prod_{k=1}^{n} s_k},$$

where E_n and M_n are defined as in equation (15) and $e_k = \begin{cases} 1 & \textit{if } i_k = j_k \neq 1 \\ 0 & \textit{otherwise} \end{cases}.$

Proof. The proof follows directly from the proofs of Propositions 2 and 3. The only difference in the induction step is that s is replaced by s_{n+1}. □

Proposition 7. *The matrices V_n and V_n^{-1} are diagonal matrices whose diagonal elements can be written as*

$$(V_n)_{ii} = (-1)^{n-W_n(\vec{i})} \prod_{k=1}^{n} \left(\frac{1}{s_k}\right)^{w_k}$$

and

$$(V_n^{-1})_{ii} = (-1)^{n-W_n(\vec{i})} \prod_{k=1}^{n} {s_k}^{w_k},$$

where

$$W_n(\vec{i}) := \sum_{k=1}^{n} w_k, \text{ with } w_k := \delta_{i_k 0}.$$

Proof. The proof follows the same steps as the proof of Proposition 4. The only difference in the induction step is that s is replaced by s_{n+1}. □

Proposition 8. *Assuming that the uncertainty or empirical error of each component of $\bar{y}_n$ is independent of the error of the other components, we can propagate the error to the estimation of the coefficients $\bar{\epsilon}_n$ as follows:*

$$\bar{\sigma}_{\epsilon_n} = \sqrt{(V_n H_n) \circ (V_n H_n)(\bar{\sigma}_{y_n} \circ \bar{\sigma}_{y_n})} \tag{33}$$

where $\bar{\sigma}_{y_n}$ and $\bar{\sigma}_{\epsilon_n}$ denote the vector of uncertainties of the phenotypes and the epistatic coefficients, respectively, and $\circ$ denotes the element-wise product.

Proof. According to equation (9) we can compute the epistatic coefficients as

$$\bar{\epsilon}_n = V_n H_n \bar{y}_n$$

This means that each ϵ is a linear combination of the elements of $\bar{y}_n$, and the coefficients of the linear combination are defined by the elements of $V_n H_n$. Writing the formula as a system of equations and using that the error propagation of a linear function is just the square root of the sum of the squares of each coefficient times the variance of each variable, we obtain:

$$\begin{aligned}
\sigma_{\epsilon_1} &= \sqrt{([V_n H_n]_{11})^2 \sigma_{y_1}^2 + \cdots + ([V_n H_n]_{1N})^2 \sigma_{y_N}^2} \\
&\vdots \\
\sigma_{\epsilon_N} &= \sqrt{([V_n H_n]_{N1})^2 \sigma_{y_1}^2 + \cdots + ([V_n H_n]_{NN})^2 \sigma_{y_N}^2}
\end{aligned}$$

which can be written with matrix notation as

$$\bar{\sigma}_{\epsilon_n} = \sqrt{(V_n H_n) \circ (V_n H_n)(\bar{\sigma}_{y_n} \circ \bar{\sigma}_{y_n})}$$

□

This equation has already been used previously in articles regarding standard $s = 2$ epistasis [2], and it also applies here for any arbitrary n and s with our definition of V_n and H_n.

For the example given in the main text regarding tRNA-ARG(CCU) with $n = 2$ and $s = 3$, we can see that applying the formula above we obtain:

$$
\begin{aligned}
\sigma_{\epsilon_{(*,*)}} &= \sqrt{\frac{\sum_k^9 \sigma_{y_k}^2}{9}} \\
\sigma_{\epsilon_{(*,1)}} &= \sqrt{\frac{\sigma_{y_1}^2 + \sigma_{y_2}^2 + \sigma_{y_4}^2 + \sigma_{y_5}^2 + \sigma_{y_7}^2 + \sigma_{y_8}^2}{3}} \\
\sigma_{\epsilon_{(1,1)}} &= \sqrt{\sigma_{y_1}^2 + \sigma_{y_2}^2 + \sigma_{y_4}^2 + \sigma_{y_5}^2}
\end{aligned}
$$

where the other terms have the same shape as the corresponding same order term above, but with different phenotypes involved. We can see that if we assume equal uncertainties the error of the epistasis coefficients increase with the order of the interaction.

References

[1] Horn R, Johnson C. Topics in Matrix Analysis. Cambridge University Press; 1991.

[2] Poelwijk FJ, Krishna V, Ranganathan R. The Context-Dependence of Mutations: A Linkage of Formalisms. PLoS Computational Biology. 2016 Jun;12(6):e1004771.
